# Decreases in Brain Reward Function Reflect Nicotine- and Methamphetamine-Withdrawal Aversion in Rats

**DOI:** 10.2174/157015911795017218

**Published:** 2011-03

**Authors:** Hisatsugu Miyata, Michio Itasaka, Naofumi Kimura, Kazuhiko Nakayama

**Affiliations:** a Department of Psychiatry, Jikei University School of Medicine, Tokyo 105-8461, Japan; b Department of Psychology, Graduated School of Humanity, Senshu University, Kanagawa 214-8580, Japan; c Department of Pharmacology, Jikei University School of Medicine, Tokyo 105-8461, Japan

**Keywords:** Nicotine, methamphetamine, intracranial self-stimulation, conditioned place aversion, brain reward system, withdrawal.

## Abstract

The purpose of the present study was to investigate whether brain reward function decreases during withdrawal from nicotine and methamphetamine, and whether decreased reward function is related to aversion during withdrawal from these drugs. For that purpose, male Sprague-Dawley rats were chronically infused subcutaneously with 9 mg/kg per day nicotine, or with 6 mg/kg per day methamphetamine using osmotic minipumps. In an intracranial self-stimulation (ICSS) paradigm, chronic infusion of nicotine and methamphetamine decreased the thresholds for lateral hypothalamic ICSS, whereas their antagonists, mecamylamine and haloperidol increased the ICSS thresholds in the rats treated with nicotine and methamphetamine, respectively. In a conditioned place aversion paradigm, mecamylamine and haloperidol produced place aversion in nicotine- and methamphetamine-infused rats, respectively. Interestingly, elevations in ICSS reward thresholds and place aversion during mecamylamine-precipitated nicotine withdrawal were almost the same in magnitude as those observed during haloperidol-precipitated methamphetamine withdrawal. The present study indicates that 1) brain reward function decreased during nicotine and methamphetamine withdrawal, and 2) a decrease in reward function may reflect the negative affective state (aversion) during withdrawal from nicotine and methamphetamine.

## 1. INTRODUCTION

Clinical evidence indicates that the affective signs of abstinence syndrome may be more relevant to drug craving and relapse to compulsive drug use than the somatic signs of withdrawal [1-3]. For that reason, the affective aspects of drug dependence have been extensively investigated using various kinds of experimental paradigms. Among them, the technique of intracranial self-stimulation (ICSS) is widely used to measure brain reward function. In animal studies, acute administration of a drug of abuse decreases ICSS reward thresholds [4, 5] and this increased sensitivity to the stimulation is considered a measure of drug-induced euphoria [6]. Furthermore, it is hypothesized that ICSS reward may be attenuated following repeated administration of a drug of abuse, resulting from neuroadapted changes of brain reward systems, and reflect dysphoria during withdrawal from the drug [7, 8]. Many studies have demonstrated elevations in ICSS reward thresholds during withdrawal from various kinds of drugs of abuse including amphetamine [9], cocaine [6], opiates [10], ethanol [11], and nicotine [12], all of which support the aforementioned hypothesis. Therefore, the present study was designed to clarify whether elevations in ICSS reward thresholds are related to the negative affective state of withdrawal, specifically focusing on two different types of psychostimulants, nicotine and methamphetamine. 

## 2. MATERIALS AND METHODS

### 2.1. Animals

Seventy-two male Sprague-Dawley rats (332-396 g) obtained from Clea Japan Inc. (Tokyo) were individually housed in an animal room at a regulated temperature (22 ± 2 ºC) with a light/dark cycle of 12/12 hours (light on at 8:00 A.M.). Each rat was fed 15 g of food per day (water freely available) throughout the experiment, except for a period of 3 days before and 7 days after surgery. This experiment was performed in accordance with the Principles of Laboratory Animal Care of Jikei University School of Medicine.

### 2.2. Drugs

(-)-nicotine hydrogen tartrate (Sigma, St. Louis, MO, USA), mecamylamine hydrochloride (Sigma), (-) methamphetamine hydrochloride (Dainipponn Seiyaku, Japan), and haloperidol hydrochloride (Sigma) were dissolved in saline and injected in a volume of 1.0 ml/kg.

### 2.3. Intracranial Self-Stimulation

#### 2.3.1. Apparatus

A standard operant chamber of 29.5 (W) x 23.5 (L) x 28.7 (H) cm (ENV-008; Med Associates, Inc., St. Albans, VT, USA) equipped with one lever and a cue light above the lever on the front wall and a house light on the rear wall was used. Sidewalls were made of transparent Plexiglas.

#### 2.3.2. Surgery

Rats were anesthetized with sodium pentobarbital (50mg/kg, i.p.) and were prepared with a stainless-steel bipolar electrode (Neuroscience, Japan) in the lateral hypothalamus (coordinates 3.8mm posterior to bregma; 1.4mm lateral to midline; 8.4mm ventral to dura) according to the atlas of Paxinos and Watson [13]. To counterbalance any possible brain asymmetries, half the rats received implants on the right side of the brain, with the other on the left side.

#### 2.3.3. Procedure

In the ICSS training sessions, a house light and a cue light were turned on and the electrical stimuli were given each time immediately after the rat pressed the lever. The stimuli consisted of 1.5 msec rectangular cathodal pulses, delivered by 100 Hz for 150 msec with a fixed current of 120 μA. Each training session lasted for 15 min. ICSS training was given at least for 6 days and continued until the number of lever press was more than 30 per min for 3 consecutive days.

To measure the baseline of ICSS responding, baseline test was performed for 15 min before each ICSS threshold test. The procedure of the baseline test was the same as in the ICSS training. An ICSS threshold test was composed of 11 time bins of 3 min separated by 1 min time out. During the time out, a house light and a cue lamp were turned off. In each test bin, these lights were turned on and the rats received the electrical stimulation after each lever press. Across the bins, the electric stimulation current was decreased by 10 μA from 120 μA to 20 μA in a descending order.

Stable baseline of ICSS responding was established for all rats before implantation of the minipumps. On day 1, an osmotic minipump (Alzet 2001, Alza Corporation, CA, USA) with a flow rate of 1.03 μl/h filled with nicotine or methamphetamine in saline was subcutaneously implanted in rats that had been anesthetized with diethylether. The concentration of nicotine and methamphetamine was adjusted for differences in body weight, but was approximately 116 and 77.3 mg/ml, resulting in continuous subcutaneous infusion at the rate of 9 mg/kg per day of nicotine and at the rate of 6 mg/kg per day of methamphetamine according to the method of a previous study [14]. The ICSS threshold test was conducted on day 2, 4, and 6 after implantation of the minipumps.

On day 7 after minipump implantation, rats received mecamylamine (0.0, 0.1, 0.5, 1.0 mg/kg, s.c.) in the nicotine- and saline-infused groups, or haloperidol (0.0, 0.1, 0.25, 0.5 mg/kg, s.c.) in the methamphetamine- and saline-infused groups, 15 min before the beginning of the ICSS threshold test session, using a within-subjects Latin-square design. Animals were required to return to baseline ICSS threshold levels for at least one ICSS session before subsequent antagonist or vehicle injections.

#### 2.3.4. Histology

Rats were sacrificed by deep anesthesia by sodium pentobarbital. The brain was removed and stored in 10% formaldehyde solution. Brain was sliced at a thickness of 100 μm and the tip of an electrode was microscopically examined.

### 2.4. Condition Place Aversion

#### 2.4.1. Appatarus

Place conditioning was conducted according to the method of Suzuki *et al.* [15, 16]. The apparatus consisted of a shuttlebox (30×60×30 cm: w×l×h) which was divided into two compartments of equal size. One compartment was white with a textured floor and the other was black with a smooth floor.

#### 2.4.2. Procedure

On day 1, rats were prepared with nicotine-, methamphetamine-, or saline-containing osmotic minipumps under the same conditions as those described for the ICSS study.

In the morning (9:00) on day 7 of nicotine or methamphetamine infusion, rats were subcutaneously injected with an antagonist of the test drug (mecamylamine or haloperidol), or saline (1.0 ml/kg), and immediately confined to one compartment of the test apparatus for 60 min. In the evening (21:00) on the same day, rats were then treated with saline or an antagonist (mecamylamine or haloperidol), respectively, and confined to the other compartment for 60 min. The pairings of injection (antagonist or saline) and compartment (white or black) were counterbalanced across all of the subjects. The control rats in the nicotine-, methamphetamine-, and saline-infused groups were injected with saline instead of mecamylamine or haloperidol in the conditioning session. After the saline injections, the rats were confined to one compartment in the morning and to the other compartment in the evening.

In the morning on day 8, tests of conditioning were performed as follows: the partition which separated the two compartments was raised to 12 cm above the floor, and a neutral platform was inserted along the seam separating the compartments. The time spent in each compartment during a 900-s session was measured automatically by an infrared beam sensor (kn-80, Natsyme Seisakusho, Tokyo, Japan).

### 2.5. Assessment of Somatic Withdrawal Signs

In the ICSS experiment, each rat was placed into a cylindrical plastic observation chamber immediately after termination of the ICSS reward threshold session following administration of mecamylamine or haloperidol, and somatic withdrawal signs were observed for 10 min. During assessment of somatic withdrawal signs, the frequency of abstinence symptoms was recorded using an opiate-abstinence scale modified to score nicotine or methamphetamine abstinence [1]. Experimenters were blind to the treatment of each rat. In the CPP experiment, somatic withdrawal signs were observed in the same manner as that in the ICSS experiment except for the fact that observation of somatic abstinence signs was conducted in the CPP apparatus.

### 2.6. Data Analysis

For the measure of the ICSS responding, number of reinforcement per min in each bin was used as the measure. On test days, the number of reinforcements at each electric current was converted into percentage of the baseline obtained on that day. To determine the ICSS threshold, S-shape curve was individually fitted according to the sigmoid-Gompertz model. Using this model, the electric current inducing 50% of baseline responding was determined as the ICSS threshold. All data were analyzed using a two-way within-subjects repeated-measures analysis of variance (ANOVA) followed by Tukey’s Studentized Range Method after observation of a statistically significant effect of treatment conditions in the ANOVA.

Conditioning scores represent the time spent in the drug-paired place minus the time spent in the vehicle-paired place and are expressed as the mean ± S.E.M. Behavioral data were statistically evaluated with a two-way repeated-measures ANOVA, which was used to determine the effects of treatment on antagonist-induced place conditioning. When the ANOVA indicated the presence of a significant effect, further analysis was conducted with Tukey’s Studentized Range Method.

## 3. RESULTS

### 3.1. ICSS Thresholds

During chronic administration, nicotine (*F *(2, 35)=5.28, *P*<0.01) and methamphetamine (*F *(2, 35)=7.62, *P*<0.01) significantly decreased ICSS reward thresholds. Individual means comparisons revealed significant effects on day 4 and day 5 of nicotine infusion (*P*<0.05), and on day 2, day 4, and day 5 of methamphetamine infusion (*P*<0.05).

As shown in Fig. (**1**), in chronic nicotine-infused and methamphetamine-infused rats, mecamylamine (*F *(1, 47)=9.59, *P*<0.01) and haloperidol (*F *(1, 47)=10.64, *P*<0.01) produced significant elevations in ICSS reward thresholds, respectively. Individual means comparisons revealed significant effects at 1.0 mg/kg mecamylamine (*P*<0.05) and at 0.25 and 0.5 mg/kg haloperidol (*P*<0.05). There was no significant effect of dose either in nicotine-infused rats (*F *(3, 47)=1.87, *P*>0.05) or in methamphetamine-infused rats (*F *(3, 47)=2.24, *P*>0.05), or treatment×dose interaction either in nicotine-infused rats (*F *(3, 47)=1.56, *P*>0.05) or in methamphetamine-infused rats (*F*(3, 47)=1.77, *P*>0.05).

### 3.2. Conditioned Place Aversion (CPA)

As shown in Fig. (**2**), the saline-control rats exhibited no preference for either compartment. Mecamylamine and haloperidol did not produce either significant place preference or place aversion in saline-infused rats. On the other hand, mecamtlamine (*F *(1, 47)=8.62, *P*<0.01) and haloperidol (*F *(1, 47)=11.28, *P*<0.01) produced place aversion in chronic nicotine- and methamphetamine-infused rats, respectively. Significant place aversion was observed at 1.0 mg/kg mecamylamine (*P*<0.01) and at 0.25 and 0.5 mg/kg haloperidol (P<0.05 and P<0.01). There was no significant effect of dose either in nicotine-infused rats (*F *(3, 47)=1.98, *P*>0.05) or in methamphetamine-infused rats (*F *(3, 47)=2.56, *P*>0.05), or treatment×dose interaction either in nicotine-infused rats (*F *(3, 47)=1.74, *P*>0.05) or in methamphetamine-infused rats (*F *(3, 47)=2.28, *P*>0.05).

### 3.3. Somatic Signs

The overall number of somatic signs did not differ between nicotine- and saline-treated rats during mecamylamine administration either in the ICSS experiment (*F *(1, 47)=2.02, *P*>0.05) or in the CPA experiment (*F *(1, 47)=1.87, *P*>0.05). Furthermore, they did not differ between methamphetamine- and saline-treated rats during haloperidol administration either in the ICSS experiment (*F *(1, 47)=1.53, *P*>0.05) or in the CPA experiment (*F *(1, 47)=2.33, *P*>0.05).

### 3.4. Histological Analysis

**Fig. (3):**
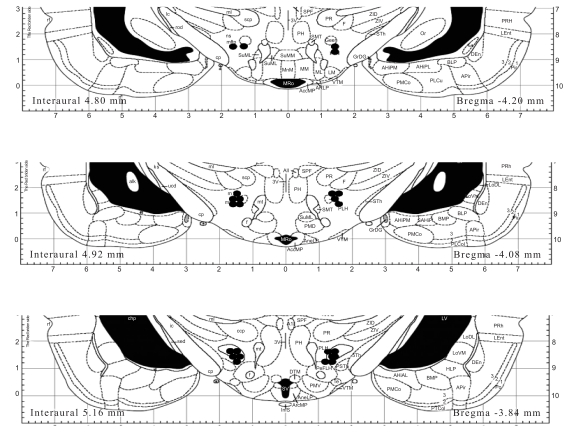
Histological localization of lateral hypothalamic stimulating electrode tips. The number beside each brain slice represents the distance from the bregma. Reconstructions based on the stereotaxic atlas of Paxinos and Watson [13]. Placements that are completely overlapping are not shown.

The results of the histological analysis indicated that the electrode tips were located in the area of the lateral hypothalamus, in an anterior/posterior range extending from -3.84 mm to -4.20 mm from the bregma. There did not appear to be any differences between the electrode locations of control and experimental animals (Fig. **3**).

## 4. DISCUSSION

The results of the current study demonstrate that chronic administration of nicotine and methamphetamine decrease ICSS reward thresholds, whereas their antagonists, mecamylamine and haloperidol increase ICSS reward thresholds and induce a CPA in rats treated with nicotine and methamphetamine, respectively. With regard to alterations in brain reward circuitry during withdrawal, it has been argued that, as dependence develops, neuroadaptations occur within the same brain circuits that mediate the reinforcing or rewarding effects of drugs of abuse following acute administration, leading to the expression of negative affective signs of withdrawal upon drug abstinence [7, 8]. Consistent with this notion, the present study indicated that nicotine as well as methamphetamine showed decreases in ICSS reward thresholds during acute administration, and increases during antagonist-precipitated withdrawal. Other drugs of abuse such as cocaine [6], opiates [10], and ethanol [11] have also been reported to induce similar pattern of effects on ICSS reward thresholds. A question as to whether such changes in brain reward circuitry are sufficient to account for the negative affective consequences of withdrawal has been under investigation. A paradigm of CPA is a useful and sensitive behavioral index to detect withdrawal aversion, as reported in previous studies involving nicotine [15, 16] and opiates [17, 10]. In the present study, mecamylamine and haloperidol induced a CPA at doses showing elevations in ICSS reward thresholds, suggesting that elevations in ICSS reward threshold may mediate aversion during withdrawal from nicotine and methamphetamine. On the other hand, mecamylamine and haloperidol failed to induce somatic withdrawal signs. Somatic signs of withdrawal from psychostimulants are known to be weaker than those from opiates, barbiturates and alcohol. Furthermore, it is more difficult to observe somatic withdrawal signs precipitated by nicotine antagonists than those elicited by spontaneous withdrawal [12]. Interestingly, in the present study, elevations in ICSS reward thresholds and place aversion during nicotine withdrawal were almost the same in magnitude as those observed during methamphetamine withdrawal, which may suggest that decreases in brain reward function, leading to withdrawal aversion, may not significantly differ in intensity between nicotine and methamphetamine, irrespective of acute effects of these drugs on the reward system. In other words, it is hypothesized that neuroadaptations in brain reward circuitry develop almost to the same levels between nicotine and methamphetamine, although they stimulate the reward system to a different degree with acute methamphetamine being stronger than acute nicotine. However, further studies are needed to clarify this question by employing a wider range of drug doses or other kinds of experimental paradigms.

In conclusion, the present study indicates that 1) brain reward function decreased during nicotine and methamphetamine withdrawal, and 2) a decrease in reward function may reflect the negative affective state (aversion) during withdrawal from nicotine and methamphetamine.

## Figures and Tables

**Fig. (1):**
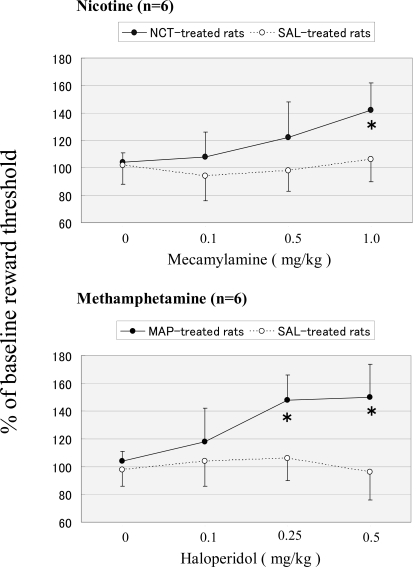
Intracranial self-stimulation reward thresholds during withdrawal precipitated by mecamylamine (upper graph) and haloperidol (lower graph) in rats that were chronically infused with nicotine and methamphetamine, respectively. Each point represents the mean percent of baseline threshold with S.E.M of 6 rats. *P<0.05 vs. saline-treated control.

**Fig. (2):**
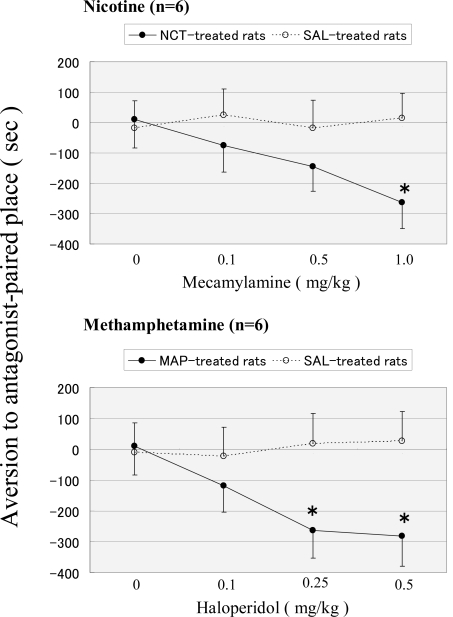
Place conditioning produced by mecamylamine (upper graph) and haloperidol (lower graph) in rats that were chronically infused with nicotine and methamphetamine, respectively. Each point represents the mean conditioning score with S.E.M. of 6 rats. *P<0.05 vs. saline-treated control.
